# Supplementary material for: Content-rich biological network constructed by mining PubMed abstracts
Source: BMC Bioinformatics. 2004 Oct 8;5:147. doi: 10.1186/1471-2105-5-147 (PMC528731; doi:10.1186/1471-2105-5-147)
Supplement: Additional File 5 — The original Chilibot query results of the term "long-term potentiation (LTP)" and 22 other terms, limiting the latest references analyzed to the years 1990, 1995, 2000, and 2004. [file 1471-2105-5-147-S5.bz2 › chilibotAdditionalFile5/ltp2000/html/SYNAPTOPHYSIN.html]

 


**SYNAPTOPHYSIN** (Input: SYNAPTOPHYSIN ) 

---


**Google Searches:** Entire Web  | EDU domain only  | PDF files only

.

**External Links:** OMIM |
LocusLink
| Swissprot
| GeneCards

  
**Maps of SYNAPTOPHYSIN**

Simple
Complete
graph in
radiant
tree
square
layout.

**New Hypothesis !**

**Synonyms** 

- synaptophysin   [PubMed]

**Synopsis**

- Our data show that synaptogyrin I and **synaptophysin** I perform redundant and essential functions in synaptic plasticity without being required for neurotransmitter release itself.  Neuron, 1999 
  [20]

- The data suggest a role for **synaptophysin** in release and indicate that activation of tyrosine kinase and **synaptophysin** phosphorylation contribute to long term potentiation  [LTP]  perhaps by modulating glutamate release.  Neuroreport, 1998 
  [20]

- In experiments in which cultured neurons were stained immunocytochemically with antibody against **synaptophysin** after electrophysiological recordings, hot spots were found to correspond to probable synaptic sites.  J Neurophysiol, 1995 
  [18]

- However, both proteins are found on membranes which are immunoisolated with antibodies directed against the synaptic vesicle membrane protein **synaptophysin**, suggesting that dynamin and p145 are localized in part on organelles which represent intermediate stages in the reformation of synapatic vesicles during recycling.  J Biol Chem, 1994 
  [16]

- After training, pp60c src from hippocampus showed enhanced interactions with synaptic proteins such as synapsin I, **synaptophysin**, and the type 2 N methyl d aspartate receptor, as well as the cytoskeletal protein actin.  Proc Natl Acad Sci U S A, 2000 
  [16]

- The action of NT 4 is accompanied by massive sprouting of axons in the vicinity of the NT 4 source as monitored by staining for acetylcholinesterase and **synaptophysin** immunoreactivity, suggesting that NT 4 may enlarge the terminal field of preganglionic nerves and enhance their access to trophic factors.  J Neurosci, 1998 
  [16]

- Analysis of another six mice from the same population that were showing weight loss and hypolocomotor activity exhibited a marked reactive gliosis as detected by immunoreactivity for glial fibrillary acidic protein and a profound loss of immunoreactivities for the presynaptic terminal vesicle marker proteins **synaptophysin** and synapsin and the dendritic marker microtubule associated protein 2 in many brain areas, but most predominantly in the cortex and hippocampus.  Neuroscience, 1999 
  [16]

- Here, we show that late phase LTP L LTP is associated with a significant increase in numbers of synaptic puncta identified by **synaptophysin** and N cadherin, an adhesion protein involved in synapse formation during development.  Neuron, 2000 
  [16]

- Parallel increases in **synaptophysin** immunoreactivity were NOT observed, thus suggesting a selective induction of synapsin I after ischemia.  Brain Res, 1999 
  [15]

- Similar expression profiles for the two proteins were observed in immunocytochemical studies of Xenopus spinal neurons in culture intense staining of **synaptophysin** was found on the first day, while synapsin I was NOT detected until after three days in culture.  Neuroscience, 1996 
  [12]

- The increase in **synaptophysin** synthesis is NOT simply the result of an increase in mRNA level, but is largely attributable to an increase in the rate of translational initiation.  J Neurosci, 1997 
  [11]

- Totest the hypothesis whether a failure to express neurotrophins or a neurotrophin receptor might underlie the pathology observed in mutant mice with degeneration of regionally distinct subpopulations of neurons, the expression of BDNF, NT 3, TrkB, TrkC and **synaptophysin** mRNA was examined in the cerebellum of mutant lurcher lc and weaver wv wv wv mice.  Brain Res Dev Brain Res, 1998 
  [10]

- Neuroscience, 1994 
  [10]

- Finally, unilateral entorhinal cortex lesions produced a marked loss of hippocampal **synaptophysin** immunoreactivity in both groups and a marked up regulation of apolipoprotein E in the wild type group.  Neuroscience, 1998 
  [10]

- SV2, synapsin I, and **synaptophysin** revealed the organization of the barrel field in the neonate.  J Comp Neurol, 1996 
  [10]
